# Stronger control-surveillance systems for vector-borne Chagas disease

**DOI:** 10.1590/0074-02760210130chgsb

**Published:** 2023-01-20

**Authors:** Rodrigo Gurgel-Gonçalves

**Affiliations:** 1 Universidade de Brasília

The multinational initiatives for the control/surveillance of Chagas launched by disease-endemic countries and the Pan American Health Organization-World Health Organization (PAHO-WHO) contributed to control house-infesting triatomine-bug populations and to reduce disease incidence. However, after 30 years, Chagas disease (CD) transmission persists the Americas. In their recent review, Rojas de Arias et al.^1^ highlight the ‘practical impossibility’ of interrupting vector-borne *Trypanosoma cruzi* transmission, due to the zoonotic nature of most transmission cycles, with well over 100 vector species widely spread across the Americas. Then, Rojas de Arias et al.^1^ emphasise the need for stronger surveillance systems to monitor and control CD. Here, I will (1) briefly discuss the prospects for interrupting vector-borne *T. cruzi* transmission and (2) provide an overview of some innovative approaches that, I believe, can play important roles in the development and operation of stronger control-surveillance systems for vector-borne CD.

Disease control needs clear goals, and those goals often heavily depend on the natural history, risk factors, transmission routes and dynamics, pathogenesis, and treatment of the disease. Eradication, elimination, reduction of incidence, reduction of the number of severe cases, and reduction of fatality rates are all possible goals of disease control programs.^2^ The most ambitious goal is disease *eradication* - the complete elimination of an infection, with no new cases recorded *in the absence of control measures*. Eradication is practically impossible for zoonoses such as CD, whose etiological agent can be transmitted by 150+ vector species and infects a wide range of wild vertebrate hosts from the USA to Patagonia.^3^ Therefore, the objective of a CD control program cannot be eradication, at least in the Americas.

The main goal of the multinational initiatives against CD was the *elimination* of the strongly synanthropic, non-native populations of a few “primary” vectors - mainly *Triatoma infestans* and *Rhodnius prolixus*.^4^ Elimination of *R. prolixus* from Mexico and some countries of Central America was certified in 2011 by PAHO/WHO, but *R. prolixus*-infested houses were detected in rural sites in Mexico after certification.^5^ Moreover, a new record of *T. infestans* in Mexico was recorded after 50 years.^6^ In Peru, non-native *T. infestans* populations were not controlled in Arequipa (see references in Rojas de Arias et al.^1^). In addition, *T. infestans* residual foci have been detected in Brazil after certification.^7^ Overall, these data indicate that elimination of non-native populations of “primary” vectors is feasible, but has many challenges.

Elimination may not be the best goal for native vectors of CD.^8^ ^,^ ^9^ There is a high species richness of Triatominae in the Americas.^3^ Moreover, it is important to highlight that the response of some native species to vector control with insecticides was not the same as that observed for non-native species. In Brazil, for example, insecticide spraying reduced dramatically *T. infestans* distribution, but no significant reduction in distribution was observed for most native species. Moreover, the interruption of chemical treatment was systematically followed by recolonisation of native species,^10^ mainly in unplastered houses.^11^ In the state of Bahia, there was a clear reduction in the distribution of *T. infestans* and an increase in the relative abundance and distribution of *T. sordida* and *T. pseudomaculata* after 40 years of the vector-control program. The high frequency of native triatomine species invading houses in the Americas in recent years^7^ ^,^ ^12^ ^,^ ^13^ highlights the need to reinforce entomological surveillance actions to prevent CD.

WHO established goals for controlling CD by 2030, including the interruption of transmission through vectorial, transfusion, transplantation and congenital routes in many endemic countries.^14^ Achieving those goals is feasible for non-vector-borne transmission,^15^ but many challenges remain for vector-borne transmission. Thus, I agree with de Rojas Arias et al.^1^ that the WHO 2030 goal of interrupting vector-borne transmission seems unfeasible because: (i) wild triatomine populations are widespread, (ii) surveillance methods for synanthropic triatomines are not perfect, and (iii) CD still has low visibility and priority. Thus, it is necessary to reinforce the surveillance of vector-borne transmission of CD. In the next paragraphs I will highlight some innovative strategies that could help strengthen this surveillance: (I) online training for health agents (II) development of apps to identify vectors and improve surveillance with community participation and citizen science, (III) vector-borne transmission-risk mapping.

I - Online training for health agents

In Brazil, the Open University of the Unified Health System (UNASUS) regularly offers online courses on infectious-disease prevention. Online courses can reach many more people than traditional courses; for example, more than 20,000 health agents have already taken the Surveillance and Control of Vectors of Importance in Public Health course (https://www.unasus.gov.br/cursos/curso/45783), which covers CD vectors and was recently revised and expanded in a Spanish version. There is also the prospect of producing an English version to reach the USA and possibly other English-speaking countries with native triatomines (e.g., Belize, Guyana or Trinidad and Tobago). Recently, another UNASUS course was opened focused on CD in primary health care (https://www.unasus.gov.br/cursos/curso/46776). The Brazilian Ministry of Health also offers courses in parasitology, chemical vector control, and triatomine-bug identification once or twice a year (R. Albuquerque, personal communication). Globally, a web-based search identified over 140 courses on vector biology and vector-borne diseases.^16^ Casas et al.^16^ conclude that vector biology courses need modernisation and suggest a worldwide unique portal for continuous training. Distance-learning courses are increasingly important for training professionals working with CD; ultimately, they strengthen control-surveillance systems and help maintain the visibility of CD.

II - Development of apps to identify vectors and improve surveillance with community participation and citizen science

An important topic in the training of heath agents working with CD is the identification of triatomines. This is because surveillance and control actions depend on which species occur in the area; the correct identification of triatomines is therefore critical for CD control-surveillance. Triatomines are traditionally identified using printed dichotomous keys based on morphological characters, but new electronic keys are available and can run on smartphones.^17^ ^,^ ^18^ TriatoKey includes 42 triatomine species recorded in Brazil and TriatoDex covers the 150 triatomine-bug species described worldwide up to 2017. TriatoDex performance was assessed in a series of blind identification tasks and correct-identification probabilities were overall high. These apps have the potential to strengthen both routine entomological activities by professional staff and CD surveillance with community participation.

Bug notification by householders enhances vector detection and can be more effective than either active searches by control agents or the deployment of vector-detection devices.^19^ One way to help householders identify triatomines in their homes would be to develop a fully-automated visual identification system. The first step towards this goal was taken by Gurgel-Gonçalves et al.^20^ The system was able to discriminate among 12 Mexican and 39 Brazilian triatomine species from high-quality, standardised dorsal photographs; incorporating distributional information raised correct identification rates to 98.9%.^21^ Another step is to test the automated identification system with lower-quality photographs taken from different angles. After incorporating a greater number of species, the system should be made available through an open-access app. The idea is to organise an automatic identification system for triatomines of the Americas in which anyone with an internet-connected smartphone can photograph a triatomine, send the picture to the system and receive the identification of the insect together with information about its importance as a vector. Another important step in this direction was the development of GeoVin (http://geovin.com.ar/), which collects geographic information on Argentinean triatomines through citizen reports of bug findings. Occurrence data (photos and geographic coordinates) are sent online, stored, and automatically integrated into the GeoVin occurrence dataset.^13^ Applications based on automated visual identification systems may substantially strengthen CD vector surveillance while promoting citizen science. Finally, community-based surveillance works best when the disease is more visible. Recent international initiatives to increase the visibility of CD include the designation of April 14 as the World Chagas Disease Day by the 72nd World Health Assembly.^22^

III - Vector-borne transmission-risk mapping

Flexible entomological-risk indicators that cover native and non-native vectors are needed to support local decision-making. The identification of areas with greater vulnerability to the occurrence of vector-borne CD is essential to prevention, control, and surveillance activities. Using ecological-risk maps based on vector distribution models and disease-risk maps based on incidence data, Sarkar et al.^23^ produced a composite map for CD risk in Texas. In Brazil, the vulnerability of municipalities to vector-mediated *T. cruzi* transmission was assessed based on socioeconomic, demographic, entomological, and environmental indicators.^24^ A similar methodological approach was used by the Brazilian Ministry of Health to analyse the vulnerability for chronic CD (file:///C:/Users/Admin/Downloads/boletim-especial-de-doenca-de-chagas-numero-especial-abril-de-2022.pdf). Another example is the recently described “TriatoScore”, an entomological-risk score that can help control-surveillance managers to assess, stratify, and manage the risk of vector-borne CD.^25^ An additional strategy is to develop an information system for disease vectors in order to plan surveillance and control activities, organise actions and integrate field and laboratory teams. The software SisVetor (https://wiki.sisvetor.com/) facilitates data collection to help control vector-borne diseases and allows the geolocation and organisation of sampling areas. SisVetor also organises surveillance activities (planning, execution, monitoring and control phases). A pilot project was installed in some Brazilian municipalities to evaluate SisVetor (R. Albuquerque, personal communication).

In summary, I fully agree with Rojas de Arias et al.^1^ when they highlight the ‘practical impossibility of interrupting vector-borne *T. cruzi* transmission’ and emphasise that stronger surveillance systems are needed to monitor and control CD. I recognise that much has been done to control CD in the Americas,^1^ ^,^ ^4^ ^,^ ^10^ ^,^ ^26^ but several major challenges must still be overcome. The good news is that innovative strategies have been developed, including online training of health agents, apps for vector identification that can improve citizen-based surveillance, or new, more flexible entomological indicators to map and manage transmission risk. I believe that the gradual incorporation of these new strategies into public-health services will improve CD control-surveillance in the Americas, and will thus help us get closer to the goal of reducing the incidence of this disease by 2030.

Comments on the article: de Arias AR, Monroy C, Guhl F, Sosa-Estani S, Santos WS, Abad-Franch F. Chagas disease control-surveillance in the Americas: the multinational initiatives and the practical impossibility of interrupting vector-borne *Trypanosoma cruzi* transmission. Mem Inst Oswaldo Cruz. 2022; 117: e210130.
